# Optimizing Hepatobiliary Lesion Assessment: The Role of Rapid On-Site Evaluation and Toluidine Blue Staining in Cytology

**DOI:** 10.7759/cureus.79287

**Published:** 2025-02-19

**Authors:** Rohan Das, Evarisalin Marbaniang, Vandana Raphael, Donboklang Lynser

**Affiliations:** 1 Pathology, North Eastern Indira Gandhi Regional Institute of Health and Medical Sciences, Shillong, IND; 2 Radiology, North Eastern Indira Gandhi Regional Institute of Health and Medical Sciences, Shillong, IND

**Keywords:** adequacy, fnac, hepatobiliary, rose, toluidne blue

## Abstract

Background: Fine-needle aspiration cytology (FNAC) is a broadly accepted method for diagnostic evaluation of pathological lesions. Rapid On-Site Evaluation (ROSE) of smears has been proven to improve the diagnostic yield of image-guided FNACs significantly. There are limited studies on the use of ROSE for image-guided FNACs from biliary tract lesions in our country.

Objective: To determine the utility and efficacy of ROSE on FNAC from neoplastic and non-neoplastic lesions of the hepatobiliary tract.

Methodology: A prospective study was conducted wherein ROSE was performed for FNACs from 50 patients with hepatobiliary tract lesions. Samples were stained with toluidine blue to check adequacy based on the proposed adequacy criteria. Cytological slides were later analyzed after staining with May-Grünwald Giemsa and Papanicolaou stains. Results were compared with those obtained from FNAC without on-site evaluation done retrospectively.

Results: From 2007 to 2016, 47 out of 330 image-guided FNACs (without ROSE) from hepatobiliary lesions were non-diagnostic. In contrast, during our study (2021-2022), only 2 out of 50 image-guided FNACs (with ROSE) were non-diagnostic. The diagnostic yield of FNAC increased from 85.76% to 96% (*P* = 0.044).

Conclusions: ROSE with toluidine blue staining significantly increases the diagnostic yield of hepatobiliary FNACs and eliminates the need for repeat procedures.

## Introduction

Fine-needle aspiration cytology (FNAC) is a well-established, safe, non-invasive, and widely accepted technique for the evaluation of various lesions. With advancements in imaging techniques (such as ultrasonography and computed tomography), even visceral lesions are now amenable and accessible to FNAC [1], including gallbladder lesions [2], which were previously inaccessible by blind FNAC procedures. Studies have shown that with the application of Rapid On-Site Evaluation (ROSE) of smears, there was a statistically significant increase in the diagnostic yield of FNACs. ROSE was found to cause a significant increase in adequacy rates [3], while also leading to a marked reduction in non-diagnostic rates [4,5]. Additionally, while performing FNACs, repeat sampling in the same sitting is preferable over a subsequent sitting, which demands more involvement of the treating physician and the operator, motivation of the patient, as well as increased expenditure [6]. Also, the total positive rate was found to be similar between 21G FNAC and 18G Tru-cut needle (TCN) biopsy procedures, but the safety of 21G FNAC was superior to that of 18G TCN. Tissues obtained by either of these two procedures were found to be sufficient for a pathological diagnosis [7]. Toluidine blue is the most cost-effective, quick, least labor-intensive, and reliable rapid stain for ROSE, especially in settings with limited resources [8].

Although there are a few studies on ROSE for hepatic malignancies, the utility of ROSE for biliary tract lesions has been poorly explored in India, especially in the Northeastern region.

We evaluated the outcome in terms of adequacy yield of FNACs conducted for hepatobiliary lesions over two different periods: January 2007 to December 2016 (retrospective study period) and April 2021 to October 2022 (prospective study period).

## Materials and methods

The study was conducted in the Department of Pathology in collaboration with the Department of Radiodiagnosis, North Eastern Indira Gandhi Regional Institute of Health and Medical Sciences (NEIGRIHMS), Shillong.

Period of study

The prospective study period was from April 2021 to October 2022 after obtaining approval from the NEIGRIHMS Scientific Advisory Committee (NSAC) and the Institutional Ethics Committee (IEC). The retrospective study period was from January 2007 to December 2016.

Study population

A total of 50 patients with hepatobiliary lesions, who fulfilled our inclusion and exclusion criteria, were included in our study prospectively. For the retrospective study period, 330 cases were included.

Inclusion criteria

All patients with radiologically or clinically diagnosed hepatobiliary tract lesions, who were advised to undergo image-guided FNAC, and attended the indoor and outdoor departments of NEIGRIHMS from 2021 to 2022, were included in the study.

Exclusion criteria

All patients with hepatobiliary tract lesions already diagnosed on biopsy, deranged blood coagulation profiles, suspected hepatic echinococcosis or hepatic surface vascular lesions were excluded from the study.

Study procedure

We conducted a prospective study in which we obtained the consent of all the patients before proceeding with FNAC. A detailed history, including family history and relevant radiological findings, was recorded. Percutaneous FNAC was performed on all patients using a Franzen needle holder under ultrasound guidance. A 22-gauge or long-length spinal needle, connected to a 20-mL disposable syringe, was used during suspended respiration. The sample received via FNAC was immediately stained with 0.5% toluidine blue for one minute and examined under a microscope at the bedside by a pathologist to check adequacy. If a sample was found to be inadequate, the FNAC procedure was repeated. A maximum of three FNAC procedures were attempted in any case. The adequate samples were categorized according to the proposed adequacy criteria (Table 1).

**Table 1:** Provisional adequacy criteria (PAC).

Inadequate	Adequate	Adequate	Adequate
Group 1	Group 2	Group 3	Group 4
Acellular smears on three needle passes	(i) Diffuse single cells or (ii) clusters containing less than or equal to 10 cells	Less than or equal to five clusters (one cluster having more than 10 cells)	More than five clusters (one cluster having more than 10 cells)

Samples deemed to be adequate were sent for routine cytological staining with May-Grünwald Giemsa (MGG) and Papanicolaou (PAP) stains. Cell block preparations were not made routinely for all cases. However, Any excess material was triaged for cell block preparation using the plasma thrombin/thrombin clot method. The cytological slides, along with the cell block section (when available), were analyzed. The results were compared with the histopathology of the lesions (if performed) or with the final diagnosis, as confirmed based on clinical presentation, radiological findings, and cytomorphological features. The results were compared with FNAC data from previous years (January 2007 to December 2016), which did not include any on-site evaluation, for statistical analysis of adequacy, sensitivity, and specificity. A non-parametric chi-square test was used to examine the diagnostic yield of FNACs across the two time periods, using the SPSS software, version 25 (IBM Corp., Armonk, NY).

## Results

**Figure 1:**
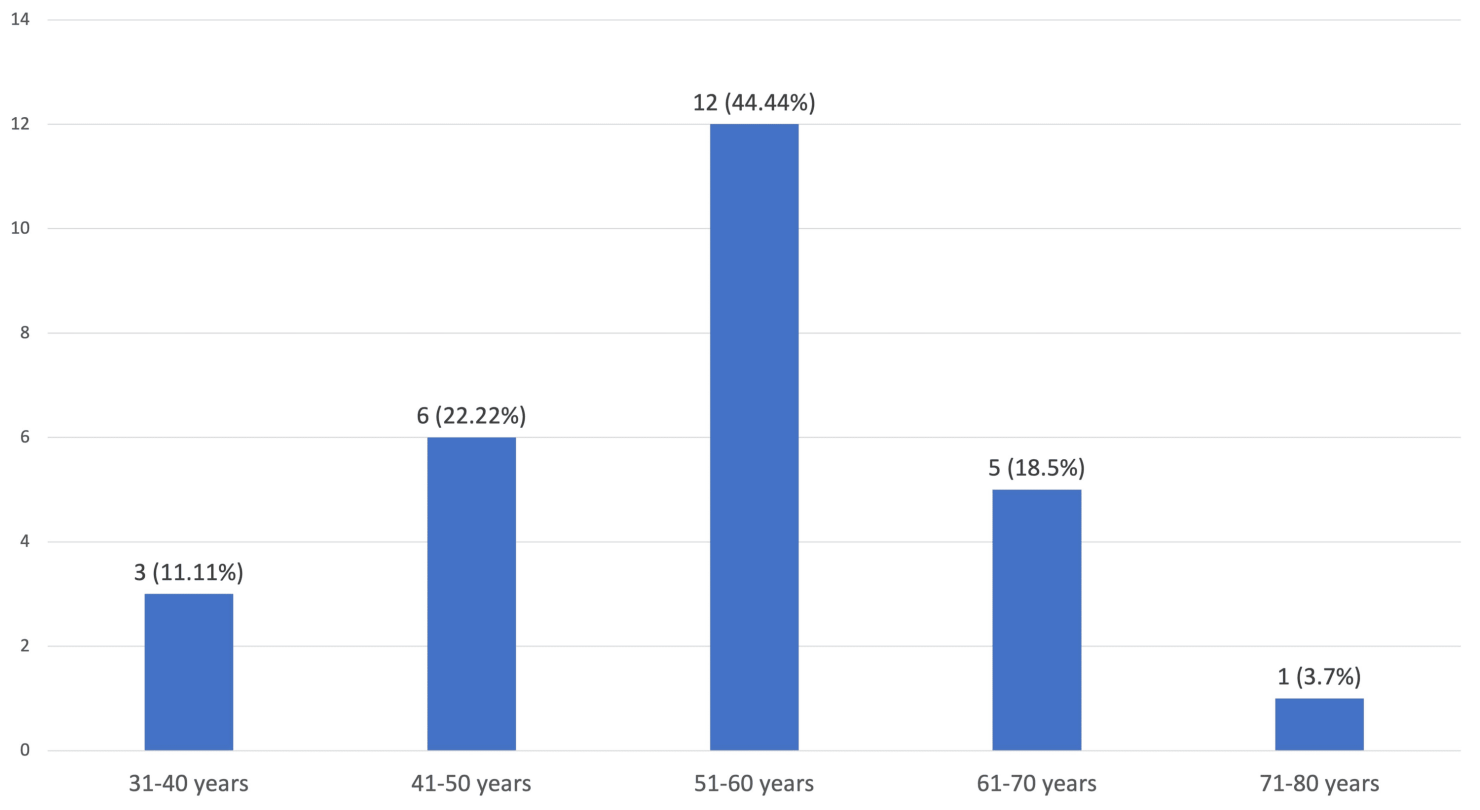
Age distribution of patients with gallbladder carcinoma.

During the prospective study period of our study (April 2021-October 2022), 50 image-guided FNACs were performed on hepatobiliary tract lesions. ROSE was performed on all of them. The patients' ages ranged from 26 to 77 years, with the median age being 53 years. Most of the patients belonged to the age group of 51-60 years, with the number of patients being 21. We received 27 patients with gallbladder carcinoma, the age distribution of which ranged from 37 to 73 years, with the median age being 55 years (mean = 55.15 years). Most of the patients belonged to the age group of 51-60 years, with the number of patients being 12 (Figure 1).

**Figure 2:**
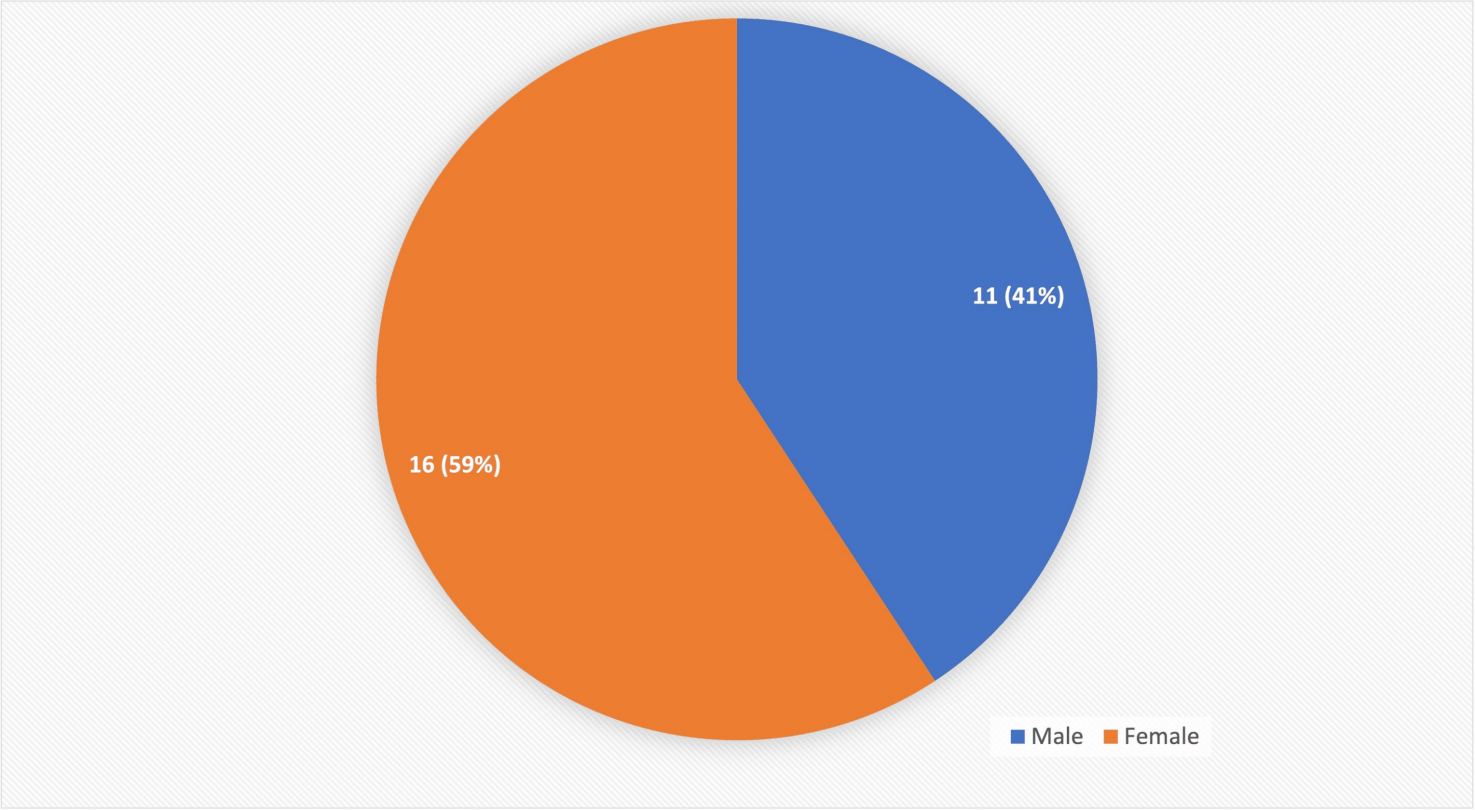
Gender distribution of patients with gallbladder carcinoma.

Out of the total 50 patients, 27 patients (54%) were found to be female and 23 patients (46%) were found to be male. Out of the total 27 patients with gallbladder carcinoma, 16 patients (59%) were found to be female and 11 patients (41%) were found to be male (Figure 2).

**Figure 3:**
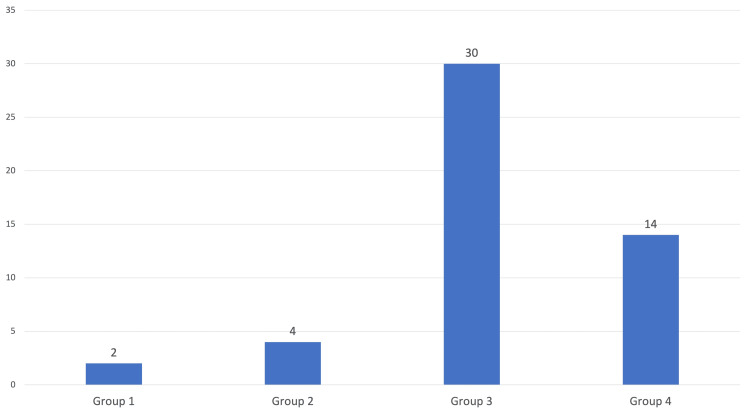
Categorization of aspirates according to PAC categories. PAC, provisional adequacy criteria

Out of the 50 aspirates, 2 (4%) were found to be inadequate even after 3 attempts and were, therefore, categorized as PAC group 1. Four (8%) aspirates were categorized as PAC group 2. Most of the aspirates, i.e., 30 (60%), were categorized as PAC group 3. Fourteen (28%) cases were categorized as PAC group 4 (Figure 3; Table 2).

**Table 2:** Correlation of PAC with the number of attempts. PAC, provisional adequacy criteria

Correlation of PAC of cases with the number of attempts	Group 4	Group 3	Group 2	Group 1	*n* (%)
Three attempts	0	1	0	2	3 (6%)
Two attempts	2	11	1	0	14 (28%)
One attempt	12	18	3	0	33 (66%)

In a study conducted at our institute during the retrospective study period [5] (January 2007 to December 2016), out of 330 FNAs (performed without onsite evaluation) taken from the gallbladder, liver, and common bile duct lesions, only 283 (85.7%) were adequate. In our study with ROSE, out of 50 aspirates, 48 aspirates were found to be adequate. There was a remarkable increase in diagnostic yield from 85.7% to 96%. This result was statistically significant, with a *P*-value of 0.044, which was less than 0.05 (Table 3).

**Table 3:** Chi-square test. ROSE, Rapid On-Site Evaluation

Diagnostic adequacy	Without ROSE	With ROSE	*P*-value	Chi-square statistic
Adequate	283	48	0.044	4.055
Inadequate	47	2		
Total	330	50		

Also, the odds ratio was calculated in this case (Table 4).

**Table 4:** Odds ratio. ROSE, Rapid On-Site Evaluation

	Adequate	Inadequate
With ROSE	48 (a)	2 (b)
Without ROSE	283 (c)	47 (d)

Odds ratio = Odds of adequacy with ROSE/Odds of adequacy without ROSE = ad/bc = 48 x 47/283 x 2 = 2256/566 = 3.99.

Thus, using the odds ratio, we can conclude that the group with ROSE had 3.99 times the odds of achieving adequacy compared to the group without ROSE. With a 95% confidence level, the confidence intervals were (0.94, 16.95). As the value 3.99 falls between the upper and lower confidence intervals, our finding is significant.

**Figure 4:**
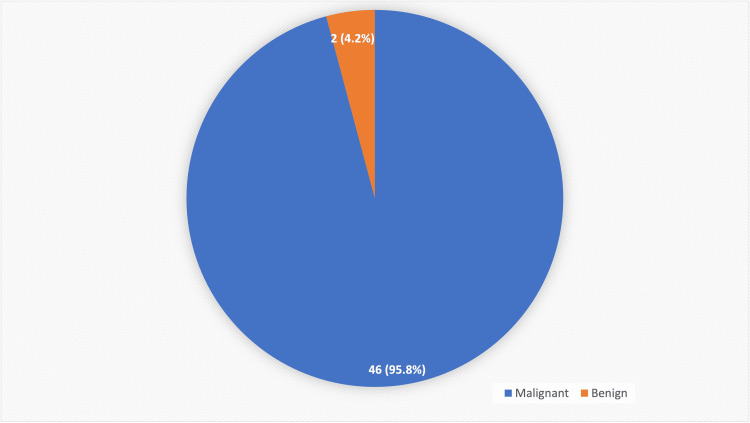
Nature of lesion.

Hence, the odds ratio was 3.99 with a 95% confidence interval. Out of the 48 adequate aspirates, 46 (95.8%) were malignant, and 2 (4.2%) were inflammatory (Figure 4).

Therefore, with ROSE, there were 46 (95.8%) malignant cases, 2 (4.2%) benign/inflammatory cases, and 2 inadequate cases even after three attempts, which were our provisional diagnoses.

**Figure 5:**
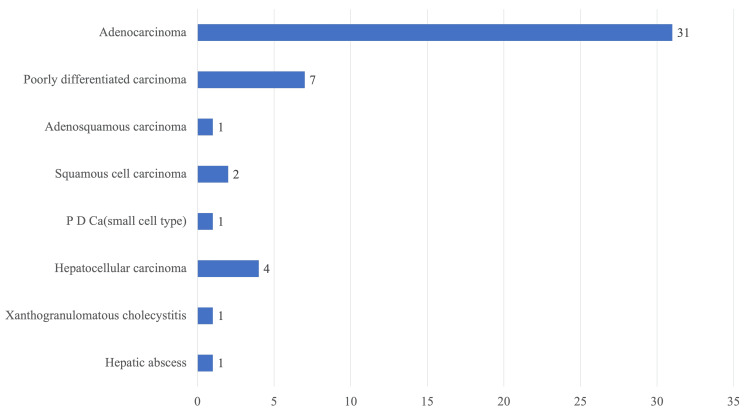
Final diagnosis based on cytomorphology. PDCa, poorly differentiated carcinoma

The final diagnosis was made after staining with MGG and PAP stains. Out of the 48 adequate cases, 31 (64.6%) were morphologically diagnosed as adenocarcinoma, 7 (14.6%) as poorly differentiated carcinoma, 1 (2.08%) as adenosquamous carcinoma, 2 (4.17%) as squamous cell carcinoma, 1 (2.08%) as poorly differentiated carcinoma (PDCa) of small cell type, 4 (8.33%) as hepatocellular carcinoma, 1 (2.08%) as xanthogranulomatous cholecystitis, and 1 (2.08%) as a hepatic abscess. Among the 27 gallbladder cancer cases, 23 (85.2%) were found to be adenocarcinomas, 3 (11.1%) were found to be poorly differentiated carcinomas, and 1 (3.7%) as adenosquamous carcinoma (Figure 5; Table 5).

**Table 5:** Correlation between provisional and final cytological diagnoses.

Provisional cytological diagnosis - *n*			Final cytological diagnosis - *n*
Adequate for malignancy - 46	Adequate for inflammatory - 2	Inadequate - 2	Adenocarcinoma - 31
			Poorly differentiated carcinoma -7
			Poorly differentiated carcinoma (small cell type) - 1
			Adenosquamous carcinoma - 1
			Squamous cell carcinoma - 2
			Hepatocellular carcinoma - 4
			Xanthogranulomatous cholecystitis - 1
			Hepatic abscess - 1

Out of the 48 adequate aspirates, 46 were diagnosed as malignant and 2 as benign on cytology. In one case of gallbladder carcinoma, the adequate cell block showed a concordant diagnosis. In four cases, including two cases of cholangiocarcinoma, one case of metastatic small cell carcinoma of the lung, and one case of xanthogranulomatous cholecystitis, needle biopsy was available and was concordant with cytology. For all other cases, the diagnosis was confirmed based on clinical features combined with the radiological picture and cytomorphological diagnosis, which informed the decisions regarding treatment made during the multi-departmental tumor board meetings.

In our study, we considered the 46 malignant cases as true positive and the two benign cases as true negative. There were no false positive cases in our study. The false negative cases were the two inadequate cases. Therefore, the sensitivity was 95.8%, specificity was 100%, positive predictive value was 100%, and negative predictive value was 50% (Table 6).

**Table 6:** Sensitivity, specificity, positive predictive value, and negative predictive value.

True positive	True negative	False positive	False negative	Sensitivity	Specificity	Positive predictive value	Negative predictive value
46	2	0	2	95.8%	100%	100%	50%

**Figure 6:**
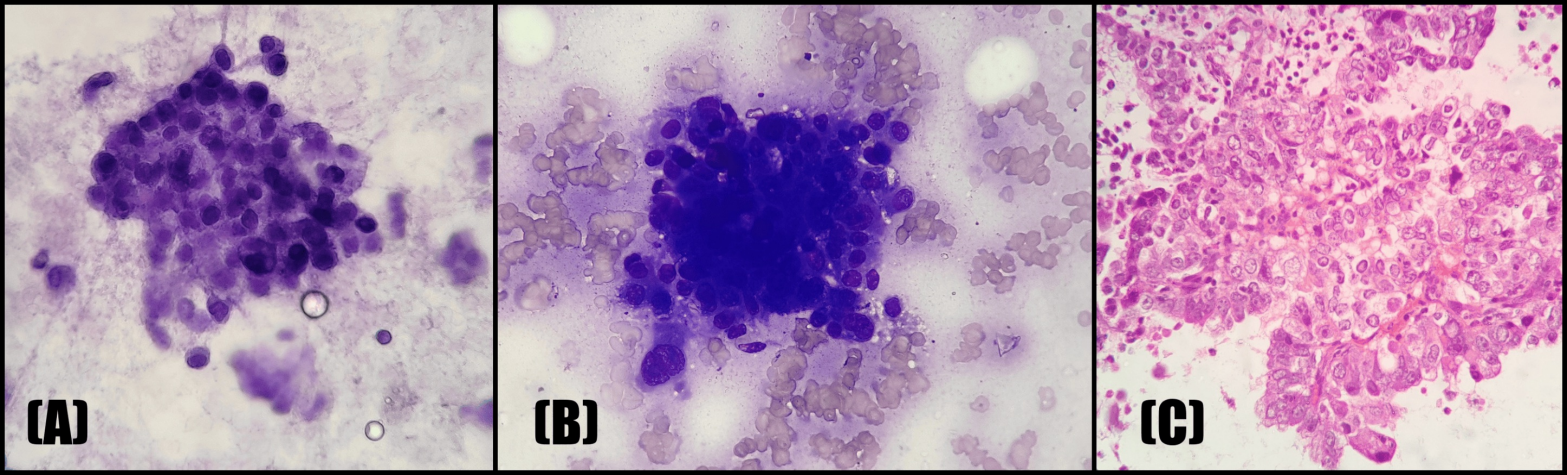
Adenocarcinoma of the gallbladder: (A) Toluidine blue stain, (B) MGG at 400x showing a cluster of malignant cells with a high N:C ratio, irregular nuclear contours, and coarse chromatin; (C) cell block, H&E at 100x showing malignant cells with a high N:C ratio and scant cytoplasm arranged in glandular and papillary patterns. H&E, hematoxylin and eosin

**Figure 7:**
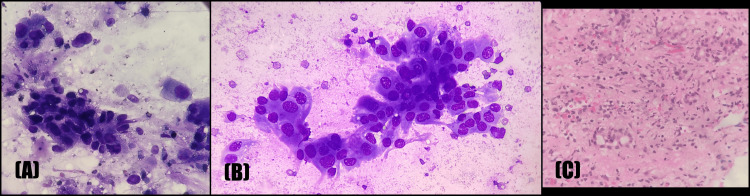
Cholangiocarcinoma: (A) Toluidine blue stain, (B) MGG at 400x showing malignant, mildly to moderately pleomorphic cells with coarse chromatin arranged in a glandular pattern; (C) H&E at 400x showing infiltrating tumor cells in a glandular pattern, with individual cells displaying mild pleomorphism. H&E, hematoxylin and eosin

**Figure 8:**
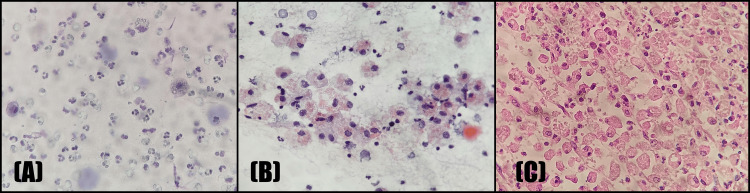
Xanthogranulomatous cholecystitis: (A) Toluidine blue stain, (B) PAP at 400x showing clustered and dispersed foamy macrophages along with inflammatory cells; (C) H&E at 400x showing sheets of histiocytes with inflammatory cells in the background. H&E, hematoxylin and eosin; PAP, Papanicolaou

**Figure 9:**
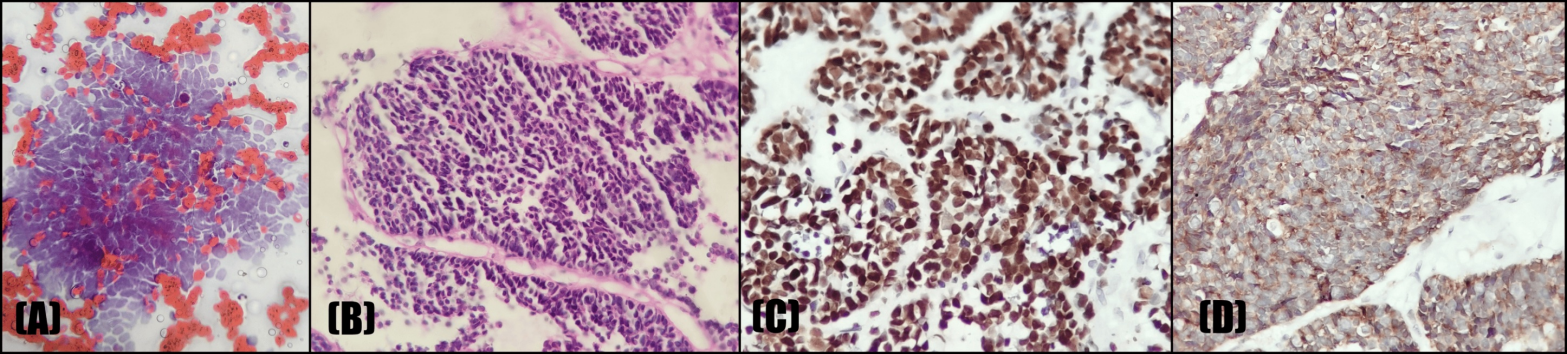
Metastatic small cell carcinoma of the lung: (A) PAP at 400x showing poorly differentiated carcinoma of small cell type with small malignant cells exhibiting molding; (B) H&E at 400x showing neuroendocrine carcinoma with malignant cells arranged in nests and evidence of molding; (C) TTF1; (D) synaptophysin: positive in tumor cells. H&E, hematoxylin and eosin; PAP, Papanicolaou; TTF1, thyroid transcription factor 1

Seven cases could not be subtyped beyond poorly differentiated carcinoma on cytomorphology (Table 7; Figures 6-9).

**Table 7:** Cases with a cytological diagnosis of poorly differentiated carcinoma.

Site of primary lesion on radiology - *n*	Site of aspiration - *n*
Gallbladder - 3	Liver - 6
Liver - 2	Gallbladder - 1
Metastatic liver lesion from adnexal primary - 1	
Bile duct - 1	

## Discussion

Adequacy of the aspirate

In a study conducted at our institute during the retrospective study period (2007-2016) [9], of the 330 FNAs performed without on-site evaluation from gallbladder, liver, and common bile duct lesions, only 283 (85.7%) were adequate. The adequacy was 81.11% for gallbladder lesions, 86.5% for liver lesions, and 100% for common bile duct lesions.

In our study with ROSE (prospective study period), 48 out of 50 aspirates were found to be adequate. There was a remarkable increase in diagnostic yield from 85.7% to 96%. Out of the two inadequate cases, one was a case of periampullary lesion with hepatic metastasis and the other was a case of breast carcinoma with hepatic metastasis. In both these cases, FNAC was done from the liver. The radiological picture in both the lesions showed multiple small deposits. Aspiration from these lesions yielded abundant blood elements, obscuring the diagnostic cells and contributing to their inadequacy (Table 8).

**Table 8:** Comparison of aspirate adequacy in both studies. ROSE, Rapid On-Site Evaluation

Diagnostic adequacy	Without ROSE	With ROSE
Adequate	283	48
Inadequate	47	2
Total	330	50
Adequacy	85.7%	96%

In a similar study by Selhi et al. [1], which compared the adequacy of hepatic FNACs between two time periods (one with ROSE and one without), a notable increase in diagnostic yield was observed, from 86.25% to 95.8%.

Correlation of provisional adequacy criteria with the number of attempts

Out of the 50 aspirates in our study, two (4%) were found to be inadequate even after three attempts. Thirty-three (66%) were found to be adequate in the first attempt. Fourteen (28%) were found to be adequate after the second attempt. One (2%) was found to be adequate after the third attempt.

In a systematic review and meta-analysis by Schmidt et al. [10], ROSE was found to increase the adequacy rate by approximately 12%, though variability was noted among studies.

Final cytological diagnosis

In our study of 48 adequate aspirates, 31 (64.6%) were found to be adenocarcinomas. Out of 27 gallbladder cancers in our study, 23 were reported as adenocarcinomas.

Of the 48 aspirates in this study, 7 (14.6%) were diagnosed as poorly differentiated carcinomas. Among the 27 gallbladder cancers, 3 were identified as poorly differentiated carcinoma.

One (2.08%) was found to be adenosquamous carcinoma of the gallbladder. Two (4.17%) were found to be squamous cell carcinoma in this study.

One case (2.08%) was diagnosed as poorly differentiated carcinoma of small cell type, while four cases (8.3%) were identified as hepatocellular carcinoma in our study.

One case was diagnosed as xanthogranulomatous cholecystitis and another as a hepatic abscess in our study. The patient with hepatic abscess had a clinical history of fever, with radiological evidence confirming the diagnosis. In the case of xanthogranulomatous cholecystitis, imaging revealed 'suspicious' gallbladder wall thickening.

We compared our findings with previous studies by Barbhuiya et al. [11], Rout et al. [12], and Selhi et al. [13], as shown in Table 9.

**Table 9:** Comparison of cytological diagnoses across various studies.

Study	Adenocarcinoma	Squamous cell carcinoma	Adenosquamous cell carcinoma	Round cell tumor	Poorly differentiated carcinoma
Barbhuiya et al. [11]	60.2%	4.8%	0.6%	2%	13.49%
Rout et al. [12]	75.47%	1.91%			
Selhi et al. [13]	87.8%	2.1%	2.1%		2.1%
This study	64.6%	4.17%	2.08%	2.08%	14.6%

Sensitivity, specificity, positive predictive value, and negative predictive value

In our study, the sensitivity for diagnosing malignancy on cytological aspirates was 95.8%, specificity was 100%, positive predictive value was 100%, and negative predictive value was 50% when the two inadequate cases were included as false negatives. When these cases were excluded from statistical evaluation, all parameters were 100%. We compared our findings with some of the findings from previous studies by Chang et al. [14], Archibugi et al. [15], and Rout et al. [12], as shown in Table 10.

**Table 10:** Comparison of sensitivity and specificity across various studies.

	Sensitivity	Specificity
Chang et al. [14]	91%	100%
Archibugi et al. [15]	74.6%	97.5%
Rout et al. [12]	98.82%	87.23%
This study	95.8%	100%

Limitations of our study

This study has several limitations. The sample size of 50 for the prospective group, while sufficient for statistical analysis, is relatively small, and a larger dataset would enhance the validity of our findings. Additionally, the focus on evaluating the adequacy of on-site smears limited the use of aspirates for cell block preparation, which is important for ancillary investigations. Most patients presented at an advanced stage, so biopsy or resection specimens were unavailable for many cases, and the final diagnosis was based on clinical presentation, radiological findings, and cytomorphology. Finally, subtyping could not be done for seven patients beyond poorly differentiated carcinoma.

## Conclusions

FNAC is a reliable technique and can be used as a substitute for invasive biopsy. Moreover, the diagnostic yield in these lesions can be increased with ROSE conducted by a pathologist using toluidine blue. ROSE also increases the likelihood of adequacy of FNAC by nearly four times compared to those without FNAC. In addition, our provisional adequacy criteria can be studied to a greater extent through multicentric studies to establish its external validity further.
